# Effect of tea polyphenols on intestinal barrier and immune function in weaned lambs

**DOI:** 10.3389/fvets.2024.1361507

**Published:** 2024-02-16

**Authors:** Yuewen Xu, Fuquan Yin, Jialin Wang, Pengxin Wu, Xiaoyuan Qiu, Xiaolin He, Yimei Xiao, Shangquan Gan

**Affiliations:** ^1^College of Coastal Agriculture Science, Guangdong Ocean University, Zhanjiang, China; ^2^The Key Laboratory of Animal Resources and Breed Innovation in Western Guangdong Province, Department of Animal Science, Guangdong Ocean University, Zhanjiang, China

**Keywords:** tea polyphenol, weaned lamb, intestinal barrier, antioxidant capacity, immune function

## Abstract

**Introduction:**

The purpose of this study was to explore the effects of tea polyphenols on growth performance, cytokine content, intestinal antioxidant status and intestinal barrier function of lambs, in order to provide reference for intestinal health of ruminants.

**Methods:**

Thirty weaned lambs (average initial weight 9.32 ± 1.72 kg) were randomly divided into five groups with six lambs in each group. The control group did not add anything but the basic diet mainly composed of Pennisetum and Corn, and the other four groups added 2, 4, 6 g/kg tea polyphenols and 50 mg/kg chlortetracycline to the basic diet, respectively. The experiment lasted for 42 days.

**Results:**

Dietary tea polyphenols improved the growth and stress response and reduced intestinal permeability of lambs (*p* > 0.05), while CTC did not affect the final lamb weight (*p* > 0.05). Both tea phenols and CTC significantly reduced inflammatory factors and enhanced the immune system (*p* > 0.05). Dietary tea polyphenols increased villus height, villus height/crypt depth, secretory immunoglobulin A (*p* > 0.05), and antioxidant enzymes, while decreasing MDA and apoptosis in the intestine (*p* > 0.05). However, compared with other groups, the content of T-AOC in jejunum did not change significantly (*p* > 0.05). Tea polyphenols also increased claudin-1 levels in the duodenum, jejunum, and ileum more than CTC (*p* > 0.05). CTC had a limited effect on the mRNA expression of *Occludin* and *ZO-1*, while tea polyphenols increased these in both the duodenum and ileum (*p* > 0.05).

**Conclusion:**

This study demonstrated that tea polyphenols can effectively improve the intestinal barrier of weaned lambs, and that they have anti-inflammatory and antioxidant effects similar to those of antibiotics. Thus, tea polyphenols could be used to replace antibiotics in ensuring safety of livestock products and in achieving the sustainable development of modern animal husbandry.

## 1 Introduction

The intestine, a critical organ for nutrient digestion and absorption, is also the largest immune organ in the body. Therefore, effective gastrointestinal function is very important for animal health, growth and production performance (1). However, when lambs are weaned, incomplete intestinal development and changes in the diet structure produce weaning stress. It leads to atrophy of the intestinal villi or deepening of crypts, resulting in a sudden drop in the capacity of young animals to digest and absorb nutrients (2). In addition, some studies have shown that weaning may promote oxidative stress, which damages the intestinal barrier (3), and can lead to intestinal cell apoptosis, intestinal immune function imbalance (4, 5), diarrhea, and weakened disease resistance (6). Therefore, alleviating the negative effects of weaning stressors has become an urgent challenge for the development of healthy animals. Antibiotics have been widely used to alleviate weaning stress (7); however, their drug residues and the development of resistance have led to a global ban on feeding large quantities of antibiotics. Therefore, researchers are exploring the use of natural plant extracts with anti-inflammatory and antioxidant properties as alternatives to antibiotics for science-based farming.

Tea, the world’s second most popular beverage after water, has seen a steady annual growth rate of approximately 4.4% over 10 years. China, as the largest producer and consumer of tea, is a rich source of tea polyphenols (8). According to its chemical structure, tea polyphenols can be divided into catechins, flavonoids, flavonols, phenolic acids and peptides, and anthocyanins, which are the general names of the polyphenols in tea. In addition, there are some other small amounts of polyphenols, such as epigallocatechin gallate, flavonoid glycosides and tannins (9). It is reported that tea polyphenols can inhibit inflammatory mediators such as cytokines, oxygen free radicals and histamine, thus contributing to the healthy growth of animals. In addition, it also has the function of stimulating signal pathways such as Nrf2, Akt and NF-κB to reduce inflammatory damage. As direct antioxidants and effective free radical scavengers, tea polyphenols can enhance the intestinal barrier function and improve the antioxidant capacity of animals, which is attributed to their phenolic hydroxyl groups (10). As a natural plant, tea polyphenols have also been proved to have the ability to regulate intestinal microbial diversity, promote intestinal health and prevent chronic metabolic diseases. In addition, many studies show that tea polyphenols have preventive effects on cardiovascular diseases, cancer, obesity, diabetes and allergic diseases (11).

Wei et al. (12) found that mice administered cyclophosphamide can recover their original level of intestinal tight junction protein and improve their antioxidant enzyme activity after being fed catechins. Studies have found that the benefits of adding green tea extract to broiler diet include increasing weight gain, improving feed conversion rate, reducing low-density lipoprotein and low-density lipoprotein cholesterol and reducing pathogenic bacteria in cecum, which is similar to the results of adding green tea by-products to pigs to improve growth performance (13, 14). In addition, tea polyphenols in monogastric animals can significantly reduce intestinal inflammation and oxidative stress, improve intestinal structure, protect intestinal mucosal health, and prevent intestinal diseases to some extent (15). However, there are few applications of tea polyphenols in ruminants; therefore, this study aimed to evaluate the effects of tea polyphenols on the intestinal morphology, intestinal barrier function, antioxidant index, immune index, and intestinal cell apoptosis of weaned lambs. Lambs were chosen as the object of study because intensive goat production is an important and expanding enterprise in China, and lambs are very susceptible to diseases. These data are necessary before considering the use of tea polyphenols as immunomodulatory interventions in clinical practice, as well as before replacing antibiotics with plant polyphenols in livestock production systems.

## 2 Materials and methods

The experimental protocol applied in this study followed the guidelines of the Animal Care and Use Committee of Guangdong Ocean University.

### 2.1 Lambs and experimental protocol

The tea polyphenols used in this experiment were provided by Xi’an Best Biotechnology Co., Ltd. (Shaanxi, China). Tea polyphenols were acquired in the form of brown powders with a special smell, in which the content of tea polyphenols is 98.1%, that of catechin is 86.6%, and that of epigallocatechin gallate is 54.2%.

Thirty healthy Leizhou black goats (about 2 months old) were selected as experimental animals, with an average weight of 9.32 ± 1.72 kg. All lambs were weaned at 2 months old, and 30 lambs were divided into 5 treatment groups with 6 replicates in each group. The experimental period was 42 days and the pre-feeding period was 7 days. The control group was fed with basic diet; the T1, T2, T3, groups were fed 2, 4, and 6 g/kg tea polyphenols, respectively; and the CTC group was fed 50 mg/kg chlortetracycline. Tea polyphenols and chlortetracycline fed every day were evenly mixed into the concentrated feed. Lambs were fed concentrate first and then roughage, and enough clean drinking water was provided every day during the experiment.

Before the experiment began, the lambs were vaccinated, deworming and numbered. Subsequently, Lamb houses, feeding pens, metabolic cages, water troughs, and feed troughs were thoroughly cleaned and sterilized. Finally, put three lambs in each pen and feed them according to the designed diet, three times a day in the morning, at noon and at night. During the feeding period, the feeding methods, experimental environment and management mode of all groups are the same. All experiments were designed by one-way random experiment. Daily teosinte was added into pellet feed by TMR, and the formulation of the basal diet (Table 1) was in accordance with the nutritional requirements of the Feeding standard of Goat, China (NY/T861-2004).

**Table 1:** Composition and level of basal diet (dry matter basis).

Items	Content
**Ingredients (%)**	
Pennisetum × sinese	50.00
Corn	29.00
Soybean meal	10.00
Wheat bran	7.50
NaCl	0.50
CaHPO_4_	0.50
Limestone	0.50
Premix^1^	2.00
Total	100
**Nutrient level**	
DM (%)	90.80
ME^2^ (MJ/kg)	10.43
Crude protein	14.69
Crude fat	2.84
ADF	26.23
NDF	39.90
Ca	0.54
P	1.10
Crude ash	7.90

^1^The premix provided the following per kg of diets: VA 17500 IU, VD6 200 IU, VE 50 IU, Cu 20 mg, Fe 75 mg, Se 0.4 mg, Mn 80 mg, Co 0.3 mg, I 1.2 mg, Zn 40 mg. DM, dry matter; ME, metabolic energy; ADF, acid detergent fiber; NDF, neutral detergent fiber. ^2^ME is the calculated value.

### 2.2 Sample collection

Blood was collected from four goat lambs in each group from the jugular vein on the day 42 of the experiment. A total of 10 mL of blood was collected from the jugular vein using a blood collection tube with coagulant, and it was then centrifuged at 3,000 rpm for 10 min. After centrifugation of the blood collection tube without anticoagulation, the supernatant was absorbed to prepare serum and then stored at −80°C for testing.

After the lamb was slaughtered, the abdominal cavity was quickly opened and duodenum, jejunum, and ileum tissue samples were collected and washed three times with pre-cooled phosphate buffer. The samples were subsequently divided into two parts. Some samples of duodenum, jejunum, and ileum samples were cut into 3–4 cm pieces, placed into an enzyme-free centrifuge tube, immediately transferred to liquid nitrogen, and stored at −80°C. In the other part, the intestinal tissues of the weaned lambs were preserved in 4% paraformaldehyde for morphological analysis and detection of the apoptosis rate.

### 2.3 Growth performance and intestinal histomorphology

On day 42, body weight and feed intake were measured, and the average daily gain (ADG), average daily feed intake (ADFI), and feed conversion rate (FCR) were calculated.

To determine the morphology of the intestinal tissue, the tissue samples were dehydrated, made transparent, embedded in paraffin, cut into 5 μm thick sections, and stained with hematoxylin and eosin (HE). The steps of the determination were as follows: (1) the fixed intestinal tissue was removed and washed with ethanol and water; (2) the tissue was dehydrated with ethanol at different concentration gradients and made transparent with xylene twice after dehydration for 20 min; (3) the tissue was soaked in wax and embedded at 60°C; (4) the wax blocks were cut into 5 μm thick slices with a slicer; (5) the wax-embedded samples were baked at 60°C, dewaxed, and dyed with HE; (6) the samples were dehydrated with ethanol, made transparent with xylene, and sealed. The tissue sections were observed using an optical microscope. ImagePro Plus 6.0 was used to measure the height of the intestinal villi (from the top of the villi to the junction of villi and crypt) and the depth of the crypt (the vertical distance from the junction of intestinal villi to the bottom of the intestinal gland), and the villus height/crypt depth value was calculated.

### 2.4 Determination of serum cortisol and immune indexes

An enzyme-linked immunosorbent assay kit (Nanjing Jiancheng Bioengineering Institute, Jiangsu, China) was used to determine the levels of immunoglobulin (IgA and IgM), COR, and cytokines (IL-1β, IL-6, IL-10, TNF-α, and IFN-γ) in the serum, and the detection method was strictly in accordance with the manufacturer’s instructions.

### 2.5 Effects of intestinal permeability and SlgA

D-lactic acid (D-LA), lipopolysaccharide (LPS), diamine oxidase (DAO), and SlgA were determined using enzyme-linked immunosorbent assay kits according to the manufacturer’s guidelines (Jiangsu Meimian Industrial Co. Ltd., Jiangsu, China).

### 2.6 Intestinal antioxidant index

After the frozen intestinal mucosa samples were thawed on ice, intestinal mucosa samples (0.5 g) were weighed and added to pre-cooled saline at a mass-to-volume ratio of 1:9 (g/mL) and ultrasonically pulverized to prepare tissue homogenates. After centrifugation at 3,000 × *g* at 4°C for 15 min, the supernatant was collected and stored in a −80°C refrigerator, and the antioxidant indices of the intestine (GSH-Px, CAT, T-SOD, MDA, T-AOC) were determined using ELISA kits according to the manufacturer’s guidelines (Nanjing Jiancheng Bioengineering Institute, Jiangsu, China).

### 2.7 Intestinal tight junction protein-related gene

Intestinal RNA was extracted using the FastPure Cell/Tissue Total RNA Isolation Kit V2 (Vazyme Biotech Co., Ltd., Nanjing, China), and then the purity and concentration of RNA were determined by spectrophotometer for subsequent experiments. cDNA synthesis was carried out through reverse transcription according to the instructions of the HiScript lI Q RT SuperMixfor qPCR (+ gDNA wiper) reverse transcription kit (Vazyme Biotech Co., Ltd., Nanjing, China). Primers were designed according to the mRNA sequences of goat target genes (ZO-1, occludin, and claudin-1) and the internal reference gene GAPDH on the NCBI official website and then passed on to Shenggong Biotechnology Co., Ltd. (Shanghai, China) for synthesis; the sequence is shown in Table 2. As shown in Table 2. The relative mRNA expression of ZO-1, occludin, and claudin-1 was determined using a real-time fluorescence quantitative PCR instrument according to the instructions of the ChamQ Universal SYBR qPCR Master Mix (Vazyme Biotech Co., Ltd., Nanjing, China), and the 2^–ΔΔCt^ method was used for calculation.

**Table 2:** Real-time PCR primer sequences.

Genes	Primer sequences (5′-3′)	GenBank accession No.	Length (bp)
*Claudin-1*	F: CCCCAGTCAATGCCAGGTATG	XM_005675123.3	169
	R: TCTTTCCCACTGGAAGGTGC		
*ZO-1*	F: TGGACAAAGAGAAGGGTGAGAC	XM_018066118.1	110
	R: TTTAGGATCACAGTGTGGTAGG		
*Occludin*	F: GGCCTCTGGGTCTCTCTACA	XM_018065677.1	154
	R: AACCATGAACCCCAGCACAA		
*GAPDH*	F: GATGCCCCCATGTTTGTGATG	XM_005680968.3	160
	R: CGTGGACAGTGGTCATAAGTC		

^1^F, forward primer; R: reversed primer. ^2^*ZO-1,* Zonula Occludens; GADPH, glyceraldehyde-3-phosphate dehydrogenase.

### 2.8 Detection of intestinal cell apoptosis

Apoptosis was assessed via a terminal deoxynucleotidyl transferase (TdT)-mediated deoxyuridine triphosphate (dUTP) nick end labeling (TUNEL) assay using an Apoptosis Detection Kit (Servare Biotech Inc., Hubei, China). First, paraffin sections were deparaffinized, and proteinase K working solution was used to cover the tissue sections for repair. The tissue sections were then incubated at 37°C for 25 min and washed with PBS. Membrane-breaking permeabilization was then performed, and the tissue was covered with drops of membrane-breaking working solution, incubated for 20 min at 27°C, placed on a shaker, and washed with PBS. The TUNEL kit was then applied, and the reagent was added to the section according to the instructions and incubated for 3 h. The sections were again covered with PBS and washed, the PBS was removed, DAPI Ran staining solution was added dropwise, and the sections were incubated in the dark for 10 min. Finally, the sections were sealed with an anti-fluorescein quencher, and images were captured through microscopic examination.

### 2.9 Statistical analysis

The experimental data were collated using Excel 2019 to establish a database, and SPSS software (version 26.0) was used for one-way ANOVA. The data were analyzed by linear effect and quadratic effect, and then the differences between groups were analyzed by Tukey multiple comparison test. The column chart is made by using GraphPad Prism 8. Statistical significance was set at *p* < 0.05.

## 3 Results

### 3.1 Effects of tea polyphenols on growth performance of weaned lambs

As shown in Table 3, the final weight of lambs in the T2 and T3 groups was significantly higher than that of lambs in the CON group (*p* < 0.05). Compared with the CON group, the ADG and ADFI of lambs in the T2, T3, and CTC groups were significantly increased, while the FCR was significantly decreased (*p* < 0.05). There were no significant differences between the other groups (*p* > 0.05). In addition, there was a significant linear and quadratic relationship (*P*_L_ < 0.05*, P*_Q_ < 0.05) between the final weight, ADG and FCR and the level of dietary tea polyphenols.

**Table 3:** Effect of dietary tea polyphenols on growth performance of weaned lambs.

Items	Groups					SEM	*p-*value		
	CON	T1	T2	T3	CTC		ANOVA	Linear	Quadratic
Initial weight (kg)	8.73	8.83	9.51	9.92	9.31	0.21	0.401	0.16	0.36
Final weight (kg)	10.24^b^	10.78^b^	12.62^a^	12.63^a^	11.38^ab^	0.35	0.01	0.01	0.02
ADG (g/d)	35.83^c^	39.88^c^	78.17^a^	69.44^ab^	53.44^b^	4.68	<0.01	<0.01	<0.01
ADFI (g/d)	524.09^b^	502.22^bc^	562.78^b^	756.67^a^	436.11^c^	30.14	<0.01	0.29	<0.01
FCR (g)	14.91^a^	12.84^ab^	7.23^d^	10.95^bc^	8.10^cd^	0.83	<0.01	<0.01	0.03

^1^CON: basal diet; T1: basal diet +2 g/kg tea polyphenols; T2: basal diet +4 g/kg tea polyphenols; T3: basal diet +6 g/kg tea polyphenols; CTC: basal diet +50 mg/kg chlortetracycline. ADG, average daily gain; ADFI, average daily feed intake; FCR, feed conversion rate. ^2^SEM, standard error of the mean. ^a–d^Values in the same row with different letters are significantly different (*p* < 0.05). Results are presented as the mean ± SEM (*n* = 6).

### 3.2 Effect of tea polyphenols on intestinal histomorphology of weaned lambs

**Figure 1:**
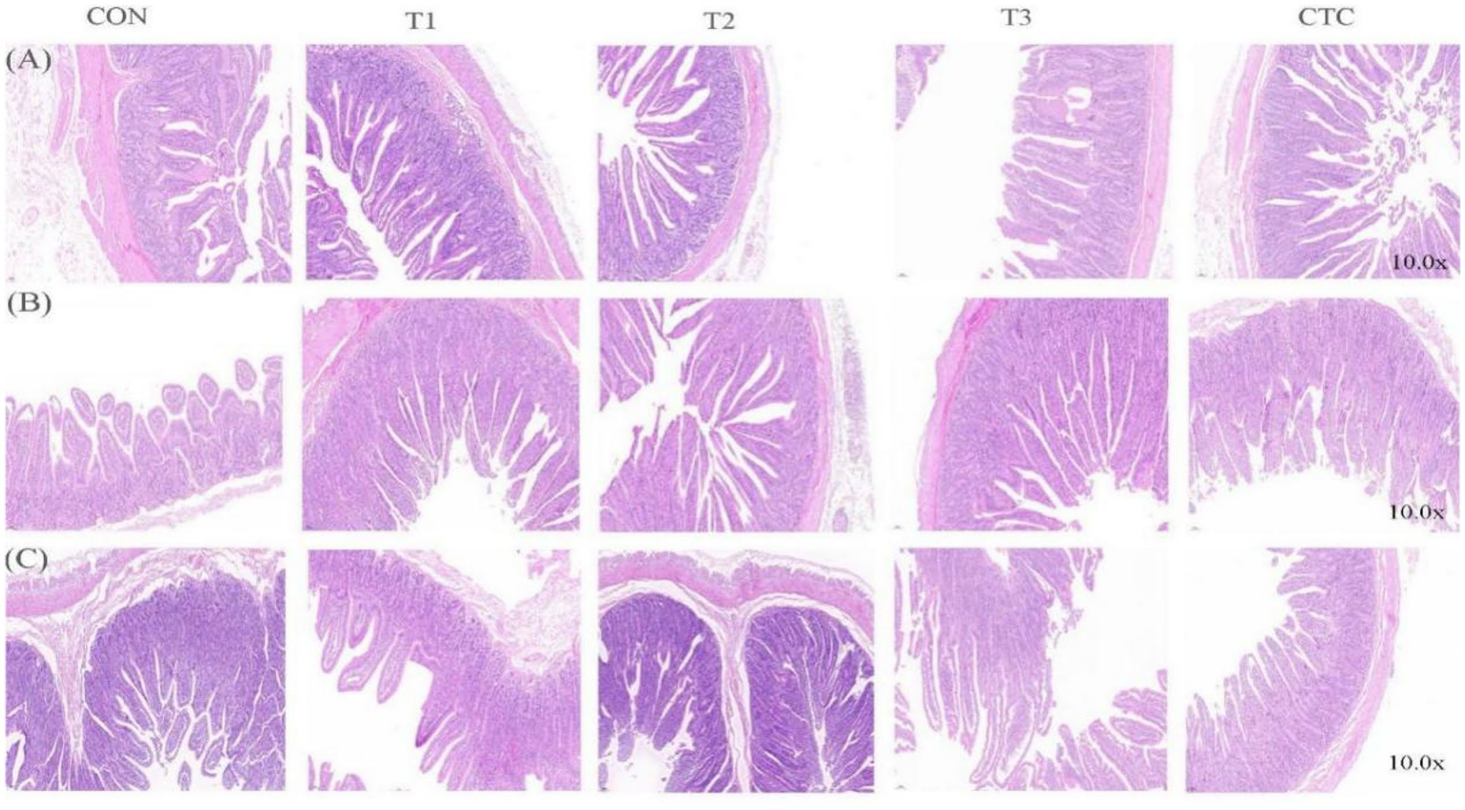
Micrographs of the duodenum, jejunum, and ileum of weaned lambs fed diets supplemented with teatoxin: **(A)** duodenum, **(B)** jejunum, and **(C)** ileum. CON: basal diet; T1: basal diet +2 g/kg tea polyphenols; T2: basal diet +4 g/kg tea polyphenols; T3: basal diet +6 g/kg tea polyphenols; CTC: basal diet +50 mg/kg chlortetracycline.

The effects of dietary tea polyphenols on intestinal morphology are shown in Figure 1 HE staining revealed that the intestinal villi in the duodenum, jejunum, and ileum were denser and longer than those in the CON group. As shown in Table 4, in the duodenum, compared with the control group, the duodenal villus height of lambs fed with tea polyphenols and CTC increased significantly, especially in T3 group of lambs (*p* < 0.05). Moreover, the duodenal CD values of lambs in T2 group were significantly lower than the other groups (*p* < 0.05), while the ratio of VH/CD was higher than the rest of the groups (*p* < 0.05). A significant linear relationship and quadratic effect between tea polyphenol concentration and duodenal intestinal histomorphology were demonstrated (*P*_L_ < 0.05, *P*_Q_ < 0.05). In addition, in the jejunum, the intestinal villi of lambs in the T3 group were significantly higher than those of the remaining groups (*p* < 0.05) and were linearly related (*P*_L_ < 0.05). There was no significant difference in jejunal CD values (*p* > 0.05), and the VH/CD ratio in the jejunum of lambs in the T2 group was significantly higher than that in the CON group (*p* < 0.05), but the addition of dietary tea polyphenols has no linear and quadratic effects on the CD value and VH/CD ratio of jejunum (*P*_L_ > 0.05, *P*_Q_ > 0.05). In the ileum, the VH values in the jejunum and ileum of the T2, T3 and CTC groups were significantly higher than those of the CON and T1 groups (*p* < 0.05), and the CD values in the ileum of the lambs in the T2 group were significantly lower than those of the CON group (*p* < 0.05), and there was a significant linear and quadratic effect between the VH and CD values and tea polyphenols (*P*_L_ < 0.05, *P*_Q_ < 0.05). The VH/CD ratios in the ileum of the lambs in the T2 group were significantly higher than those in the other groups (*p* < 0.05), but there was only a quadratic effect among the test treatment groups (*P*_Q_ < 0.05).

**Table 4:** Effect of dietary polyphenols on intestinal morphology of weaned lambs.

Items	Groups					SEM	*p-*value		
	CON	T1	T2	T3	CTC		ANOVA	Linear	Quadratic
**Duodenum**									
VH (μm)	479.13^c^	619.55^b^	652.87^b^	827.63^a^	711.65^b^	28.70	<0.01	<0.01	0.04
CD (μm)	421.35^a^	426.43^a^	278.48^b^	510.68^a^	477.15^a^	21.22	<0.01	0.04	0.01
VH/CD	1.14^c^	1.51^bc^	2.35^a^	1.63^b^	1.49^bc^	0.10	<0.01	0.04	<0.01
**Jejunum**									
VH (μm)	495.75^c^	583.85^bc^	661.15^ab^	816.23^a^	696.08^ab^	31.07	<0.01	<0.01	0.08
CD (μm)	393.80	482.8	375.35	514.23	489.25	19.92	0.142	0.128	0.912
VH/CD	1.28^b^	1.22^b^	1.87^a^	1.61^ab^	1.44^ab^	0.08	0.07	1.83	0.08
**Ileum**									
VH (μm)	457.15^b^	559.23^b^	615.28^ab^	778.68^a^	535.70^b^	31.24	<0.01	0.03	<0.01
CD (μm)	417.87^ab^	460^ab^	347.08^b^	554.13^a^	508.25^a^	22.74	0.02	0.04	0.35
VH/CD	1.13^b^	1.23^b^	1.81^a^	1.44^ab^	1.08^b^	0.09	0.03	0.80	<0.01

^1^CON: basal diet; T1: basal diet +2 g/kg tea polyphenols; T2: basal diet +4 g/kg tea polyphenols; T3: basal diet +6 g/kg tea polyphenols; CTC: basal diet +50 mg/kg chlortetracycline. VH, villus height; CD, crypt depth; VH/CD, ratio of villus height to crypt depth. ^2^SEM, standard error of the mean. ^a–c^Values in the same row with different letters are significantly different (*p* < 0.05). Results are presented as the mean ± SEM (*n* = 6).

### 3.3 Effects of tea polyphenols on serum cortisol and immunoglobulin in weaned lambs

**Figure 2:**
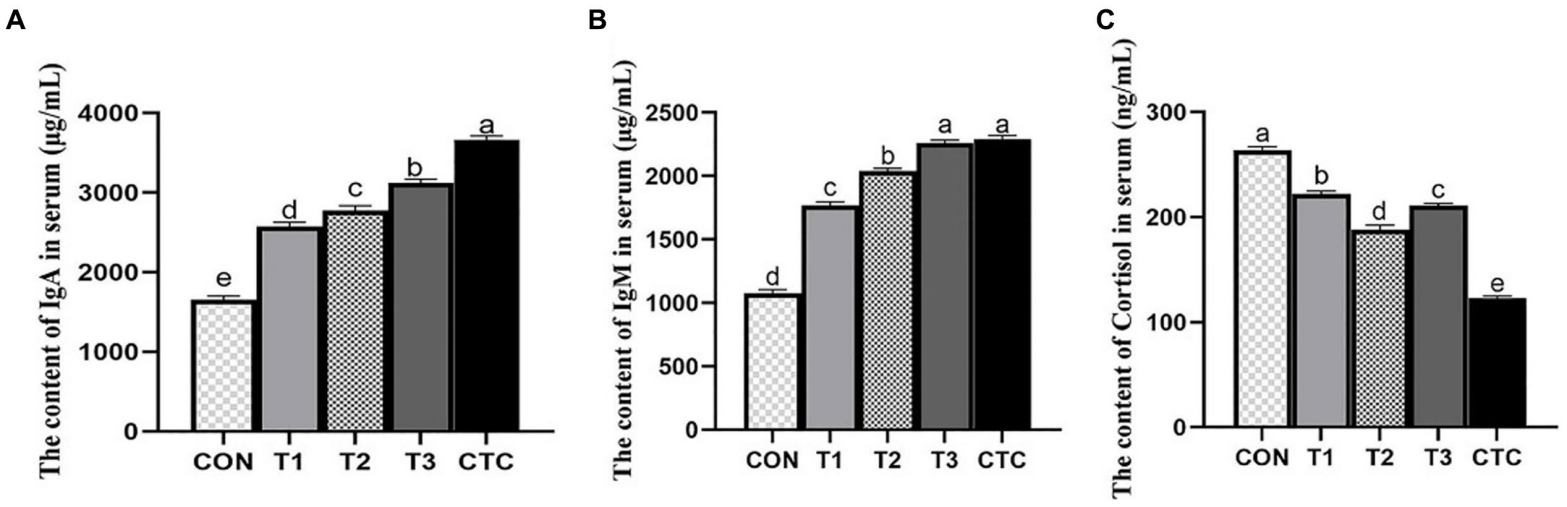
Effects of tea polyphenols on serum cortisol hormone and immune globulin of weaned lambs. ^1^CON: basal diet; T1: basal diet +2 g/kg tea polyphenols; T2: basal diet +4 g/kg tea polyphenols; T3: basal diet +6 g/kg tea polyphenols; CTC: basal diet +50 mg/kg chlortetracycline. **(A–C)** The content of IgA, IgM, and cortisol in the serum, respectively. IgA, immunoglobulin A; IgM, immunoglobulin M. ^2^SEM, standard error of the mean. ^a–e^Values in the same row with different letters are significantly different (*p* < 0.05). Results are presented as the mean ± SEM (*n* = 6).

As shown in Figure 2, IgA and IgM in the serum of lambs in T1, T2, T3 and CTC groups were significantly higher than those in the control group (*p* < 0.05), and the immunoglobulin content in CTC group was higher than that in other groups (*p* < 0.05). Serum cortisol levels in lambs were significantly lower in all tea polyphenol groups, especially in the T2 group (*p* < 0.05). The serum cortisol content of lambs in the CTC group was significantly lower than that of the other groups (*p* < 0.05).

### 3.4 Effects of tea polyphenols on cytokines in serum of weaned lambs

**Figure 3:**
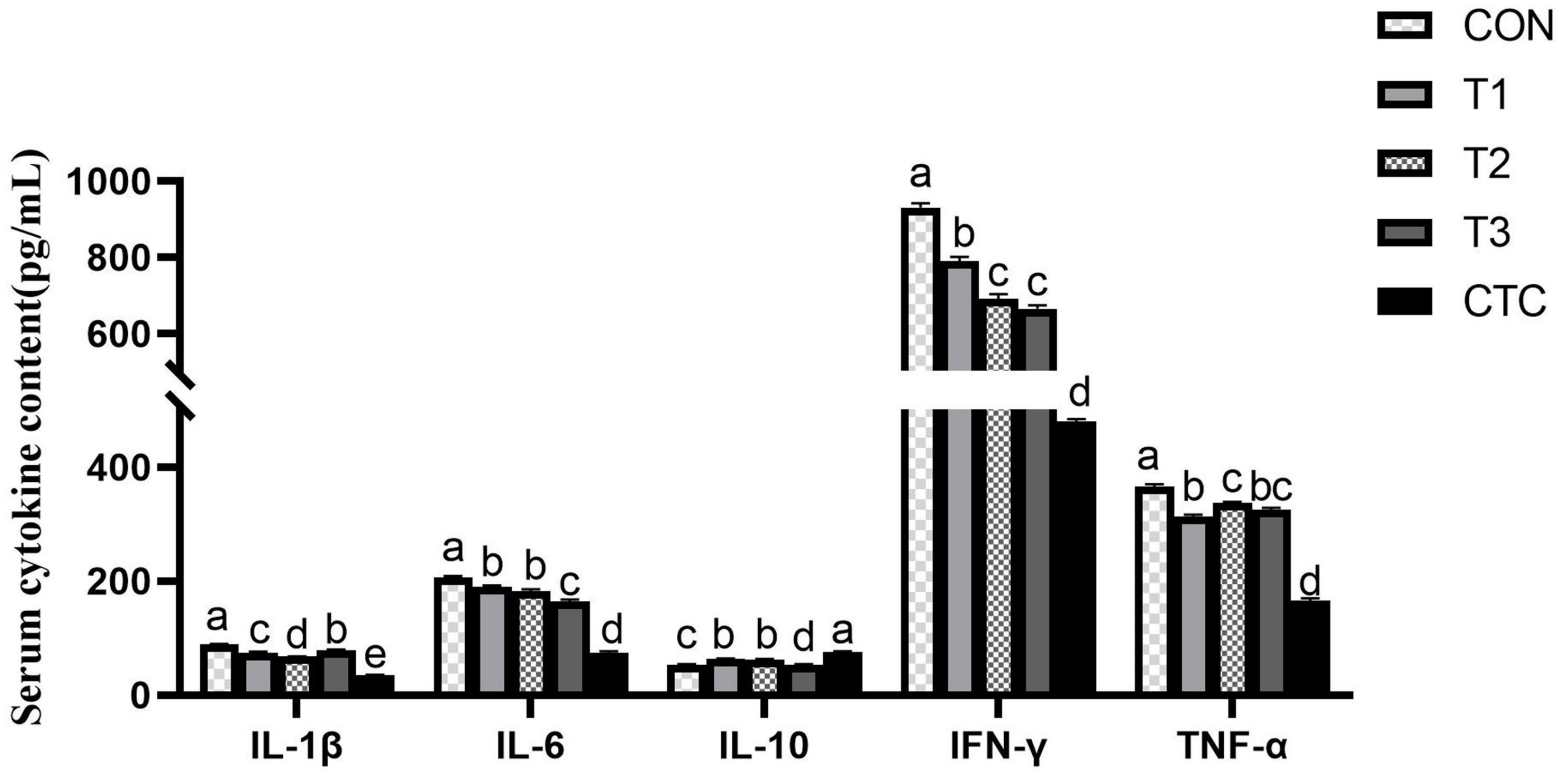
Effect of tea polyphenols on cytokines in serum. ^1^CON: basal diet; T1: basal diet +2 g/kg tea polyphenols; T2: basal diet +4 g/kg tea polyphenols; T3: basal diet +6 g/kg tea polyphenols; CTC: basal diet +50 mg/kg chlortetracycline. IL-1β, interleukin-1β; IL-10, interleukin-10; IL-6, interleukin-6; TNF-α, tumor necrosis factor-α; IFN-γ, interferon-γ. ^2^SEM, standard error of the mean. ^a–d^Values in the same row with different letters are significantly different (*p* < 0.05). Results are presented as the mean ± SEM (*n* = 6).

In order to further evaluate the anti-inflammatory effect of tea polyphenols, we measured the cytokines which are very important to indicate the inflammatory state of animals, and studied whether adding tea polyphenols can improve the immune function of weaned lambs. The results are displayed in Figure 3. Compared with the control group, all groups of lambs supplemented with tea polyphenols and CTC significantly reduced the levels of cytokines IL-1β, IL-6, TNF-α and IFN-γ in serum (*p* < 0.05), and significantly increased the serum levels of IL-10 (*p* < 0.05), especially in lambs inT3 and CTC groups.

### 3.5 Effects of tea polyphenols on intestinal permeability of weaned lambs

**Figure 4:**
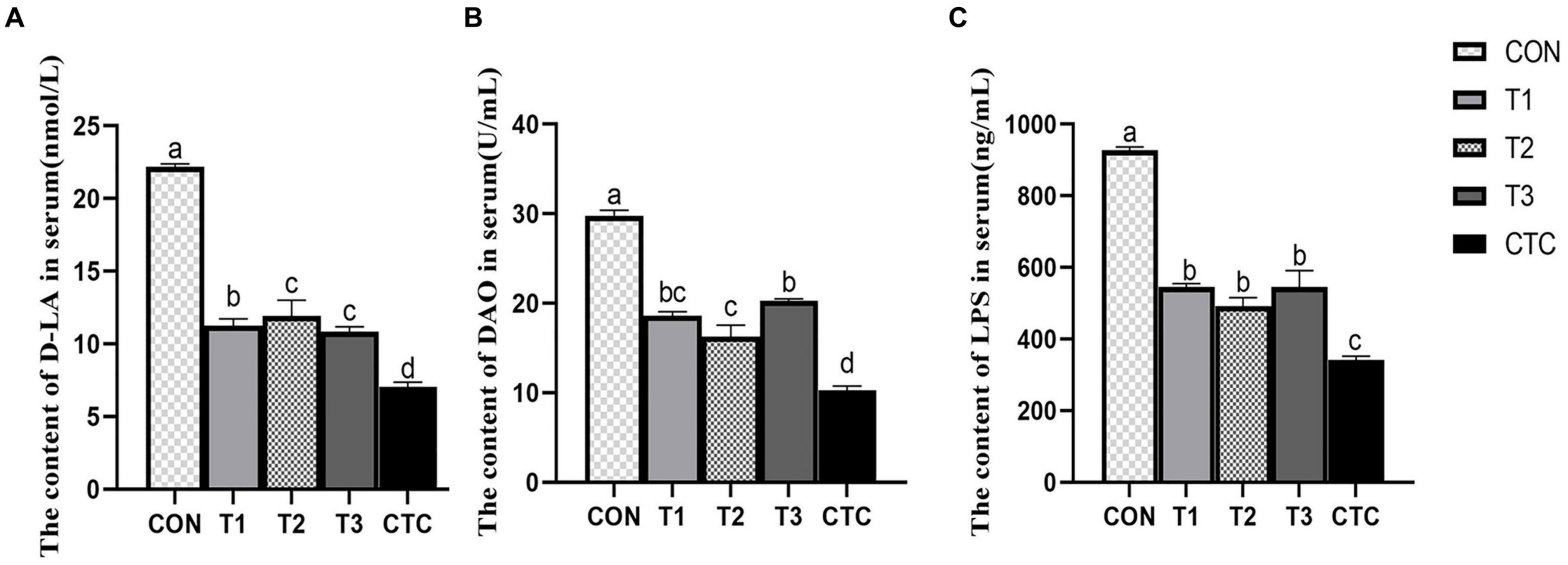
Effect of dietary tea polyphenols on intestinal permeability in weaned lambs. ^1^CON: basal diet; T1: basal diet +2 g/kg tea polyphenols; T2: basal diet +4 g/kg tea polyphenols; T3: basal diet +6 g/kg tea polyphenols; CTC: basal diet +50 mg/kg chlortetracycline. **(A–C)** The content of D-LA, DAO, and LPS in the serum, respectively. D-LA, D-lactic acid; DAO, diamine oxidase; LPS, lipopolysaccharide. ^2^SEM, standard error of the mean. ^a–d^Values in the same row with different letters are significantly different (*p* < 0.05). Results are presented as the mean ± SEM (*n* = 6).

The contents of D-LA, LPS and DAO in lamb serum were significantly decreased by adding different concentrations of tea polyphenols and antibiotics (*p* < 0.05), thus affecting intestinal permeability. Results As shown in Figure 4, the serum levels of d-lactic acid, endotoxin and DAO in the CON group were significantly higher than those in the other groups (*p* < 0.05). In the tea polyphenols group, the contents of D-LA, LPS and DAO in serum of lamb in T2 group were significantly lower than those in other tea polyphenols groups (*p* < 0.05). The contents of D-LA, endotoxin and DAO in serum of lambs in CTC group were significantly lower than those in other groups (*p* < 0.05).

### 3.6 Effect of tea polyphenols on the content of SlgA in intestine of weaned lambs

**Figure 5:**
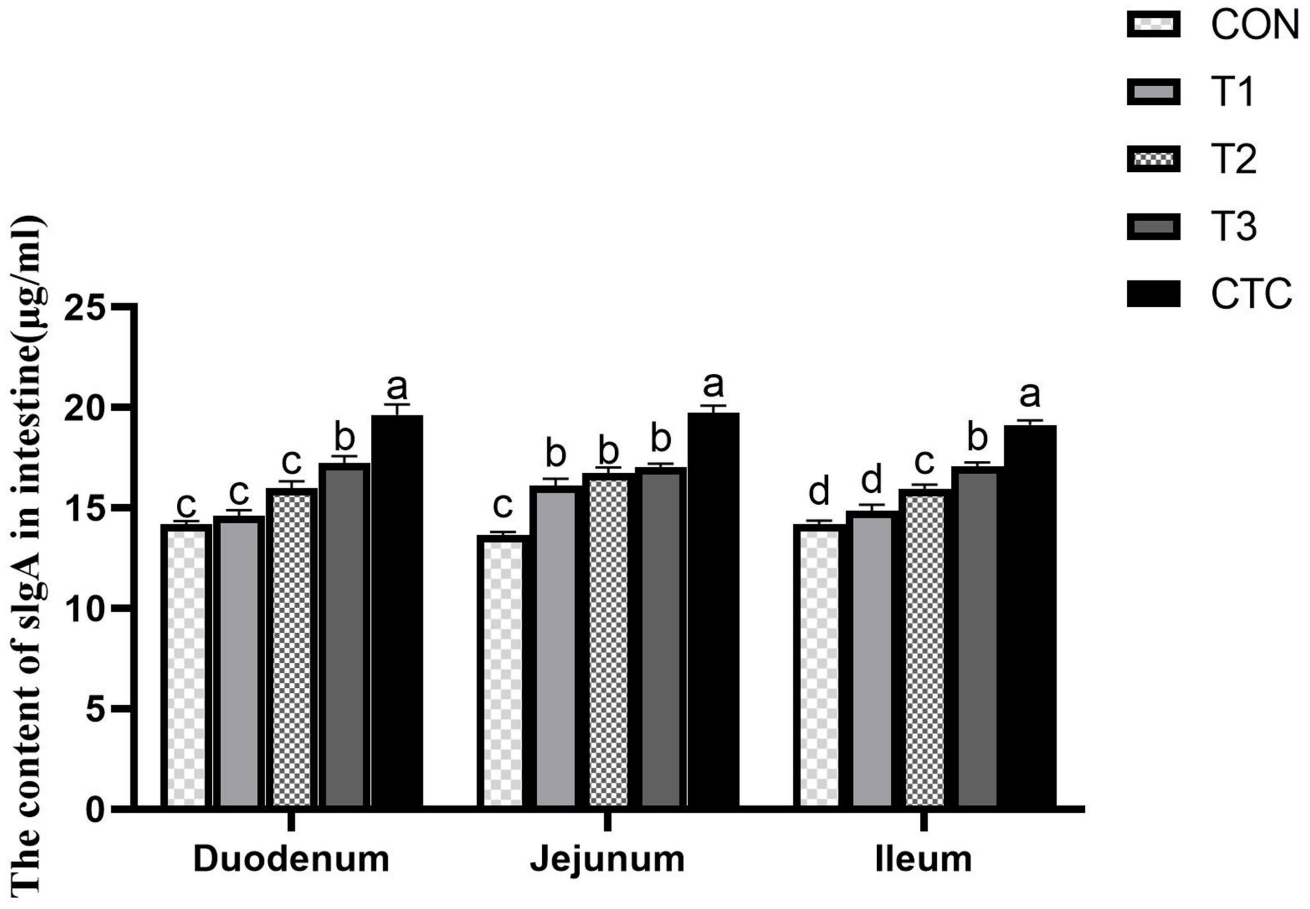
Effect of dietary tea polyphenols on intestinal SlgA content in weaned lambs. ^1^CON: basal diet; T1: basal diet +2 g/kg tea polyphenols; T2: basal diet +4 g/kg tea polyphenols; T3: basal diet +6 g/kg tea polyphenols; CTC, basal diet +50 mg/kg chlortetracycline. SlgA, secretory immunoglobulin A. ^2^Results are presented as the mean ± SEM (*n* = 6). ^a–d^Means were significantly different (*p* < 0.05).

The effects of different concentrations of tea polyphenols on intestinal SlgA content are shown in Figure 5. In the duodenum, the SlgA content in lambs in the T3 and CTC groups was significantly higher than that in the other groups (*p* < 0.05). In the jejunum, the SlgA content in the other groups was significantly higher than that in the CON group (*p* < 0.05). In the ileum, compared with the CON group, the SlgA content in lambs in the T2, T3, and CTC groups was significantly higher (*p* < 0.05). In particular, the SlgA content of lambs in the CTC group was higher than that of the remaining groups (*p* < 0.05).

### 3.7 Effect of tea polyphenols on intestinal antioxidant capacity of weaned lambs

**Figure 6:**
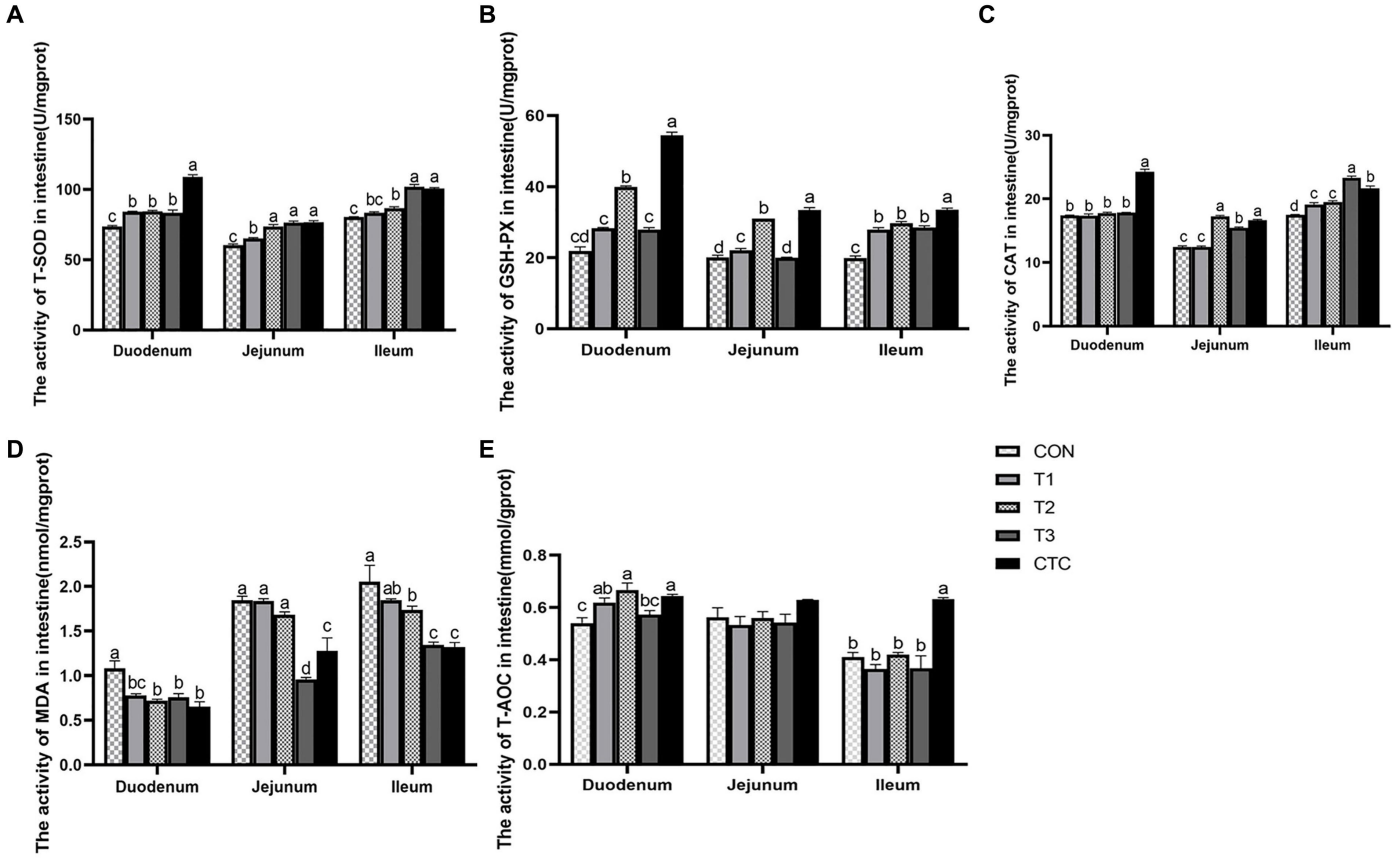
Effect of tea polyphenols on intestinal antioxidant power in weaned lambs. ^1^CON: basal diet; T1: basal diet +2 g/kg tea polyphenols; T2: basal diet +4 g/kg tea polyphenols; T3: basal diet +6 g/kg tea polyphenols; CTC: basal diet +50 mg/kg chlortetracycline. **(A–E)** The activity of T-SOD, GSH-PX, CAT, MDA, and T-AOC in the duodenum, jejunum, and ileum, respectively. T-SOD, total superoxide dismutase; CAT, catalase; GSH-PX, glutathione peroxidase; MDA, malondialdehyde; T-AOC, total antioxidant capacity. ^2^SEM, standard error of the mean. ^a–d^Values in the same row with different letters are significantly different (*p* < 0.05). Results are presented as the mean ± SEM (*n* = 6).

Figure 6 shows that in the duodenum, compared with CON group, the activity of T-SOD in the intestines of lambs in all tea polyphenols groups and CTC groups increased significantly (*p* < 0.05), and the content of MDA in the intestines of lambs in T2, T3 and CTC groups decreased significantly (*p* < 0.05). The GSH-PX activity of lambs in T2 and CTC groups was significantly higher than that in other groups (*p* < 0.05), and the T-AOC activity of lambs in T1, T2 and CTC groups was significantly higher than that in CON and T1 groups (*p* < 0.05). In addition, the CAT content of lamb in CTC group was significantly higher than that in other groups (*p* < 0.05), and there was no significant difference among other groups (*p* > 0.05). In jejunum, the T-SOD activity of lambs in T2, T3 and CTC groups was significantly higher than that in CON and T1 groups (*p* < 0.05). In addition, the activities of GSH-PX and CAT in lamb intestines in T2 and CTC groups were significantly higher than those in other groups (*p* < 0.05), and the MDA content in lamb intestines in T3 and CTC groups was significantly lower than that in other groups (*p* < 0.05). Compared with the control group, there was no significant change in T-AOC (*p* > 0.05). In the ileum, the activity of T-SOD increased gradually with the increase of tea polyphenol concentration, especially in T3 and CTC groups were significantly higher than the rest of the groups (*p* < 0.05). The ileal GSH-PX activity of the lambs in CON group was significantly lower than the rest of the groups and the GSH-PX activity of the lambs in CTC group as significant (*p* < 0.05). The CAT activity of lambs was significantly higher in all tea polyphenol groups compared to CON group and was significantly higher in T3 group than the rest of the groups (*p* < 0.05). In addition, the intestinal MDA of lambs in T2, T3 and CTC groups was significantly lower than that of CON and T1 groups. The intestinal T-AOC of lambs in CTC group was higher than that of the remaining groups (*p* < 0.05), and the differences among the rest were not significant (*p* > 0.05).

### 3.8 Effect of tea polyphenols on genes related to intestinal tight junction protein in weaned lambs

**Figure 7:**
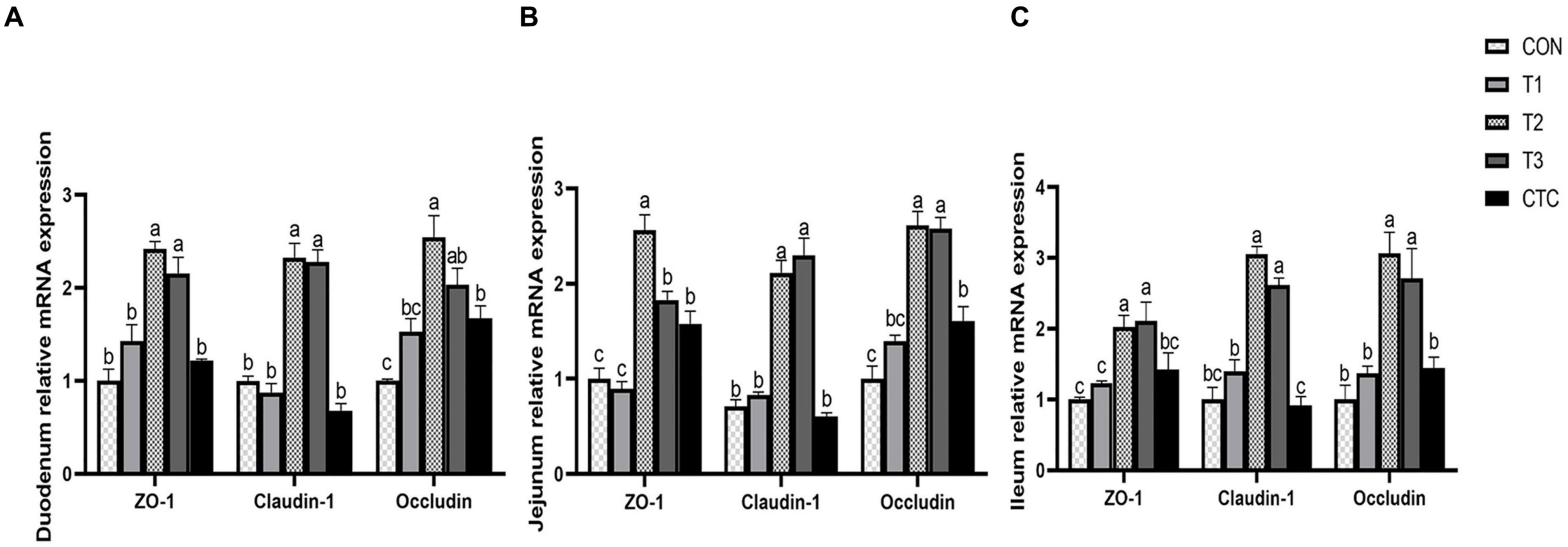
Effects of tea polyphenols on intestinal tight junction proteins in weaned lambs. ^1^CON: basal diet; T1: basal diet +2 g/kg tea polyphenols; T2, basal diet +4 g/kg tea polyphenols; T3, basal diet +6 g/kg tea polyphenols; CTC, basal diet +50 mg/kg chlortetracycline. **(A–C)** The gene expression of ZO-1, claudin-1, and occludin in the duodenum, jejunum, and ileum, respectively. ^2^The results are expressed as the mean ± SEM (*n* = 6). The mean values of ^a–d^ were significantly different (*p* < 0.05).

Except for the histological observations, the integrity of intestinal epithelium is usually determined by the expression of key tight junction genes. Thus, we further measured the relative gene expression of ZO-1, Occludin, and Claudin-1 in in sheep intestine and the results were listed in Figure 7. In duodenum, the contents of *ZO-1*, *Claudin-1* and *Occludin* in intestines of lambs in T2 and T3 groups were significantly higher than those in CON group (*p* < 0.05), and the contents of *Occludin* in intestines of lambs in CTC group were also significantly higher than those in CON group (*p* < 0.05). In jejunum tissue, the contents of *ZO-1*, *Claudin-1* and *Occludin* in the intestines of lambs in T2 and T3 groups were significantly higher than those in CON group and F1 group, while the contents of *ZO-1* and *Occludin* in the intestines of lambs in CTC group were significantly higher than those in CON group (*p* < 0.05). In ileum tissue, the contents of *ZO-1*, *Claudin-1* and *Occludin* in lamb intestine in T2 and T3 groups were significantly higher than those in other groups (*p* < 0.05), but there was no significant difference between CTC group and other groups (*p* > 0.05).

### 3.9 Effect of tea polyphenols on apoptosis rate of intestinal cells in weaned lambs

**Figure 8:**
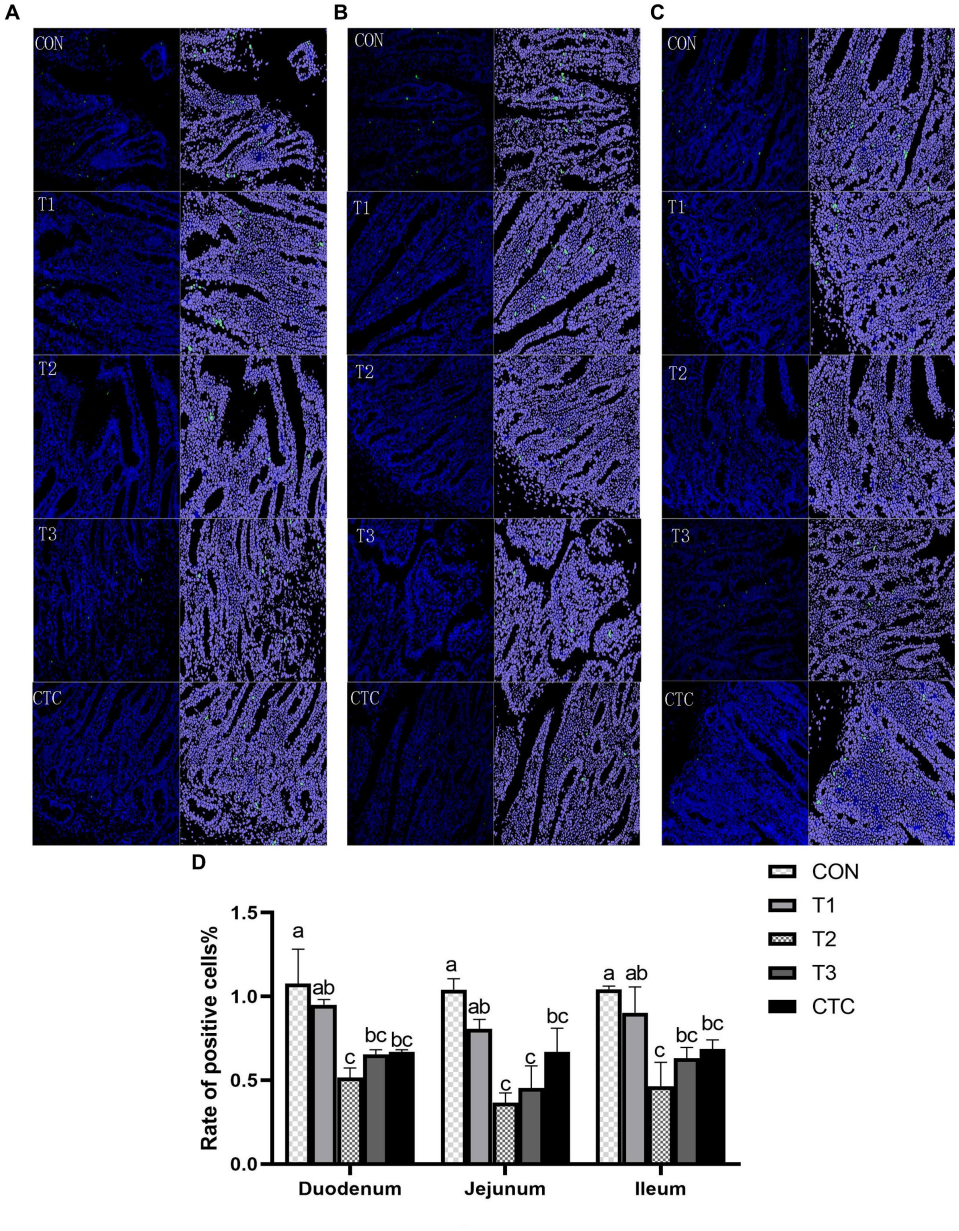
Effects of tea polyphenols on apoptosis in the intestines of lambs. Representative small intestine cross-sections (200×) of lambs from CON, T1, T2, T3, and CTC groups after TUNEL staining. Cells green in color are apoptotic cells, and those in blue are nonapoptotic cells. **(A)** Duodenum, **(B)** jejunum, and **(C)** ileum. **(D)** Apoptotic cell death rate and the results are expressed as SEM ± mean (*n* = 6); ^a–c^Means are significantly different (*p* < 0.05). CON: basal diet; T1: basal diet +2 g/kg tea polyphenols; T2, basal diet +4 g/kg tea polyphenols; T3, basal diet +6 g/kg tea polyphenols; CTC, basal diet +50 mg/kg chlortetracycline.

As shown in Figure 8, the apoptosis rates of lambs in the duodenum, jejunum, and ileum in the T2, T3, and CTC groups were significantly lower than those in the CON group (*p* < 0.05). The differences between the remaining groups were not statistically significant (*p* > 0.05).

## 4 Discussion

### 4.1 Effects of tea polyphenols on growth performance and intestinal morphology of weaned lambs

It has been demonstrated that intestinal structure and development play a crucial role in the digestive and absorptive processes of the intestines and that intestinal villi have an absorptive function, which can be indicated by villus height and villus surface area (16). The depth of crypt determines the speed of epithelial cell formation by mitosis of intestinal villi, and reflects the colonization rate and maturity of crypt cells. Therefore, the value of villus height/crypt depth is an important index to measure intestinal development and function (17, 18). In addition, some studies have shown that there is a positive correlation between villi length, nutrient absorption and digestibility. The shorter the intestinal villi and the deeper the crypt, the weaker the digestibility, thus affecting the growth potential of animals (19). Early weaning induces villus atrophy and crypt hypertrophy, resulting in diminished nutrient digestion and absorption, which may precipitate diarrhea (20). Aziz-Aliabad et al. (21) found that adding green tea powder to broiler feed significantly increased villus height and villus height/crypt depth values, enhanced the digestion and absorption of nutrients, and improved growth performance. Another study found that feeding fermented tea dregs to juvenile largemouth bass resulted in higher intestinal villus heights and widths, indicating, demonstrating, enhanced intestinal integrity and stability (22). Furthermore, Yang et al. (23) compared the effects of different levels of green tea by-products (0.5%, 1%, and 2%) with those of antibiotics and obtained significant results in terms of body weight gain. These results are similar to those of the present study. In this study, the addition of 4 g/kg tea polyphenols significantly reduced the recess depth of the duodenum and ileum and significantly improved the growth performance and intestinal villus height of the animals. Tea polyphenols can improve intestinal development by enhancing the integrity of the intestinal mucosa’s morphology, thus improving animal feed conversion rates and the digestion and absorption of nutrients, which ultimately improves animal growth performance.

### 4.2 Effects of tea polyphenols on cortisol and immune function of weaned lambs

The immune function of the intestinal mucosa is mediated by immune cells and cytokines. When animals are stimulated by weaning, macrophages can produce and secrete a large number of inflammatory cytokines, including IL-1β, IL-6, IFN-γ, and TNF-α, which mediate the inflammatory response in animals (24). Immunoglobulin is the main antibody involved in mucosal immunity; it can specifically bind antigens to exert immune effects and it plays an important role in humoral immunity. An increase in immunoglobulin secretion indicates an improvement in immune function (25). COR is typically used as an index to measure stress in animals. Related studies have shown that when lambs are in the weaning stage, the serum skin COR increases significantly over a short time (26), and LPS of Gram-negative bacteria can activate TLR4-mediated inflammatory pathways, such as NF-κB, and induce the production of inflammatory cytokines (IL-1β, IL-2, IL-6, and TNF-α) (27). The addition of green tea polyphenols to juvenile Wuchang bream under ammonia stress significantly reduced the cortisol concentration, and the IL-1B, TNF-α, and IgM levels in the spleen also significantly decreased, thus alleviating oxidative stress and ammonia damage (28), which was similar to the results of this study that tea polyphenols significantly reduced the levels of cytokines and cortisol in serum and increased the content of immunoglobulin. Song et al. (29) found that oolong tea polyphenols inhibited the activation of NF-κB in mice, downregulated the levels of pro-inflammatory cytokines (TNF-α, IL-1β, and IL-6), and significantly increased the levels of the anti-inflammatory factor IL-10. In addition, administering Chinese sweet tea to mice with induced allergies resulted in increased fecal IgA levels, indicating enhanced intestinal immunity (30). Studies have shown that 4–6 g/kg tea polyphenols can play an anti-inflammatory role by regulating the transduction of cytokines, reducing the content of pro-inflammatory cytokines, and maintaining the health of the body by increasing the immunoglobulin and IL-10 contents.

### 4.3 Effect of tea polyphenols on antioxidant capacity of weaned lambs

In the process of weaning, because the intestine is the organ with the largest contact area with food, it is stimulated by various stressors, such as foreign antigens, microorganisms, and their toxins in the diet, and excessive free radicals are produced in the body. When the antioxidant defense system of the body cannot remove excessive free radicals (ROS), the oxidation-reduction homeostasis of the body is unbalanced, leading to oxidative stress (31). When excessive oxygen free radicals are produced, they cause damage to lipids and cell membranes, proteins, and nucleic acids, resulting in intestinal damage (32, 33). The antioxidant activity of polyphenols depends on their ability to react with ROS to a great extent, and the o-quinone or p-quinone methylated substance derived from polyphenols can also upregulate the gene of the antioxidant reaction element, thus improving the enzyme activities of CAT, SOD and GPx, restoring redox stability, and preventing systemic or local inflammation (34). Ding et al. (35) found that adding tea residue to the basic diet can significantly improve the activities of GSH-Px, T-AOC, CAT, and SOD activities in the serum of growing pigs and reduce MDA content. MDA is the main product of lipid peroxidation, it can be used to monitor the lipid oxidation state. Studies have found that 300 mg/kg of tea polyphenols can reduce the MDA concentration in the intestines of young hybrid sturgeons (36). Similarly, it has been reported that catechin can alleviate the oxidative damage to chicken lymphocytes induced by hydrogen peroxide, increase the expression of antioxidant genes SOD and GSH-Px, and demonstrate the antioxidant ability of scavenging free radicals and reducing the formation of H_2_O_2_ and ROS (37). These experimental results are similar to the results of this study in that adding 4–6 g/kg tea polyphenols and antibiotics were found to improve the antioxidant activity in the intestines of weaned lambs, significantly reduce the MDA content, and improve the antioxidant capacity of the body. Therefore, tea polyphenols have antioxidant properties. In addition, the improvement in antioxidant capacity may be closely related to the improvement in intestinal integrity and immunity observed in this study.

### 4.4 Effect of tea polyphenols on intestinal permeability and barrier function of weaned lambs

Intestinal permeability refers to the ability of the intestinal mucosal epithelium to allow some types of molecules to pass through it in a passive diffusion mode that is not mediated by a carrier or channel. An increase in intestinal permeability inevitably leads to the invasion of bacteria and endotoxins from the intestinal cavity into the rest of the body through the intestine, potentially resulting in severe infections (38). In addition, when the intestinal tract is damaged, the D-LA produced by bacterial metabolism and cleavage accumulates excessively, and the intracellular enzyme DAO existing in intestinal villous epithelial cells crosses the intestinal mucosal barrier and enters the blood circulation together with D-LA (39). Therefore, its serum content can reflect the integrity of intestinal barrier function and the degree of injury (40). In addition, the intestinal epithelial tight junction structure composed of proteins such as Claudins, Occludin and ZO-1 forms a selectively permeable intercellular barrier by sealing the intercellular space between adjacent intestinal epithelial cells, preventing environmental toxins, intestinal cavity antigens, and microorganisms from entering the circulatory system (41). Studies have shown that green tea extract can improve the intestinal barrier function of high-fat mice, alleviate the decrease in tight junction protein *ZO-1* and *Claudins* genes, and reduce the endotoxin content in the serum, thus providing some protection against intestinal damage (42). The administration of catechin, the main component of tea polyphenols, to mice can also reduce the activity of serum DAO and the concentration of D-LA, while also increasing the abundance of Occludin and ZO-1 in the duodenum, jejunum, and ileum, which further indicates that catechin supplementation can maintain the integrity of the intestinal barrier in mice (43). In addition, adding L-theanine to the diet can significantly increase the mRNA and protein expression of *ZO-1*, *Claudin-1*, and *Occludin* in the jejunum, ileum and epithelial cells of piglets, and decreased their serum levels of D-LA and DAO (44). These results are similar to the results of this study, which demonstrated that adding 4 mg/kg of tea polyphenols can reduce intestinal permeability and increase the mRNA expression of tight junction proteins. This shows that tea polyphenols protect the integrity of the intestinal barrier in weaned lambs and are beneficial to their intestinal health.

### 4.5 Effects of tea polyphenols on apoptosis rate and SlgA of intestinal cells in weaned lambs

Apoptosis plays an important role in programmed cell death and cell division and is closely related to animal health. Many factors, such as inflammation, can induce the apoptosis of intestinal cells, leading to the destruction of mucosal integrity and changes in epithelial barrier function, which in turn can result in the invasion of bacterial pathogens (45, 46). Intestinal inflammation and injury caused by weaning stress trigger the release of reactive substances, such as nitric oxide and oxygen, into the intestinal cavity, leading to intestinal epithelial cell apoptosis and diarrhea (47). Tea polyphenols can inhibit exogenous cell injury, such as oxidative stress, reduce cell apoptosis in tissues, and ensure the health of the body (48). SlgAis the main component of the intestinal mucosal defense system, which can inhibit the attachment of microorganisms to the respiratory epithelium and the reproduction of pathogenic microorganisms, as well as maintain the internal environment of the intestinal mucosa (49). Studies have shown that feeding apple polyphenols to pigs can increase intestinal SlgA production to enhance intestinal immunity and improve body health (50). In addition, Yuan et al. (51) showed that feeding vanadium to chickens increases the apoptosis rate of chicken duodenal cells and causes gastrointestinal disorders, but adding tea polyphenols could inhibit this increase in the apoptosis rate, or even make it decline. Studies have shown that DSS can induce colitis, causing oxidative stress and apoptosis in mice. TUNEL analysis has further demonstrated that DSS can significantly increase the frequency of apoptotic cells in the colon mucosa, but green tea polyphenols have been found to greatly reverse this trend and improve mucosal barrier function (52) In this study, 4 and 6 g/kg of tea polyphenols and chlortetracycline had a good inhibitory effect on intestinal cell apoptosis in weaned lambs and increased the content of SlgA in the intestine, indicating that tea polyphenols could enhance the intestinal mucosal immunity of weaned lambs and inhibit the apoptosis caused by weaning stress, which was similar to the findings of Ma et al. (53) and Giakoustidis et al. (54) in mice. Therefore, tea polyphenols can reduce the oxidative stress of the intestine during weaning, reduce the damage caused by free radicals to epithelial cells, and thus protect the morphology of the intestine.

## 5 Conclusion

The results showed that the effect of adding 4–6 g/kg tea polyphenols to the diet was better, which could not only enhance the immunity and antioxidant capacity of lambs, but also improve the intestinal barrier function, reduce intestinal damage and protect intestinal health. The potential mechanism of tea polyphenols may be closely related to inhibiting intestinal inflammation, oxidative stress and reducing apoptosis. These findings provide theoretical support for developing tea polyphenols as feed additives to improve the intestinal health of livestock.

## Data availability statement

The datasets presented in this study can be found in online repositories. The names of the repository/repositories and accession number(s) can be found in the article/supplementary material.

## Ethics statement

The animal studies were approved by the Animal Care and Use Committee of Guangdong Ocean University (SYXK-2018-0147, 2018). The studies were conducted in accordance with the local legislation and institutional requirements. Written informed consent was obtained from the owners for the participation of their animals in this study.

## Author contributions

YuX: Conceptualization, Data curation, Formal analysis, Investigation, Methodology, Software, Visualization, Writing – original draft. FY: Funding acquisition, Methodology, Resources, Validation, Writing – review & editing. JW: Software, Writing – review & editing. PW: Software, Writing – review & editing. XQ: Investigation, Writing – review & editing. XH: Formal analysis, Writing – review & editing. YiX: Formal analysis, Writing – review & editing. SG: Conceptualization, Methodology, Project administration, Resources, Validation, Writing – review & editing.
